# MiR-182 promotes cancer invasion by linking RET oncogene activated NF-κB to loss of the HES1/Notch1 regulatory circuit

**DOI:** 10.1186/s12943-016-0563-x

**Published:** 2017-01-26

**Authors:** Alf Spitschak, Claudia Meier, Bhavani Kowtharapu, David Engelmann, Brigitte M. Pützer

**Affiliations:** 1 Institute of Experimental Gene Therapy and Cancer Research, Rostock University Medical Center, Schillingallee 69, 18057 Rostock, Germany; 2 Current address: Department of Ophthalmology, Rostock University Medical Center, Rostock, Germany

**Keywords:** miR-182, HES1, RET, Tumor progression, Medullary thyroid cancer

## Abstract

**Background:**

Dominant-activating mutations in the RET proto-oncogene, a receptor tyrosine kinase, are responsible for the development of medullary thyroid carcinoma (MTC) and causative for multiple endocrine neoplasia (MEN) type 2A and 2B. These tumors are highly aggressive with a high propensity for early metastasis and chemoresistance. This attribute makes this neoplasia an excellent model for probing mechanisms underlying cancer progression.

**Methods:**

The expression level of miR-182 was measured in MTC tumor specimens and in TT cells by real-time RT-PCR. TT cells and modified NThy-ori 3.1 that stably express RETM918T were used to investigate RET-dependent regulation of miR-182. Identification and validation of miR-182 targets and pathways was accomplished with luciferase assays, qRT-PCR, Western blotting and immunofluorescence. In vitro, overexpression and knockdown experiments were carried out to examine the impact of miR-182 and HES1 on invasion and migration.

**Results:**

We found that miR-182 expression is significantly upregulated in MTC patient samples and tumor-derived cell lines harboring mutated RET. Inhibition of RET oncogenic signaling through a dominant-negative RET∆TK mutant in TT cells reduces miR-182, whereas overexpression of RETM918T in NThy-ori 3.1 cells increases miR-182 levels. We further show that overexpression of this miRNA in NThy.miR-182 cells promotes the invasive and migratory properties without affecting cell proliferation. MiR-182 is upregulated after RET induced NF-κB translocation into the nucleus via binding of NF-κB to the miR-182 promoter. Database analysis revealed that HES1, a repressor of the Notch pathway, is a target of miR-182, whose upregulation correlates with loss of HES1 transcription in MTC tissue samples and mutant RET cell lines. Moreover, we demonstrated that the 3′UTR of the HES1 mRNA bearing the targeting sequence for miR-182 clearly reduced luciferase reporter activity in cells expressing miR-182. Decreased expression of HES1 promotes migration by upregulating Notch1 inhibitor Deltex1 and consequent repression of Notch1.

**Conclusion:**

We demonstrate a novel mechanism for MTC aggressiveness in which mutated RET/NF-κB-driven expression of miR-182 impedes HES1 activation in a negative feedback loop. This observation might open new possibilities to treat RET oncogene associated metastatic cancer.

**Electronic supplementary material:**

The online version of this article (doi:10.1186/s12943-016-0563-x) contains supplementary material, which is available to authorized users.

## Background

Medullary thyroid carcinoma (MTC) is a rare neuroendocrine tumor that originates from calcitonin secreting parafollicular C cells of the thyroid gland and accounts for approximately 3% of thyroid cancers [1]. The key cause for the development of MTC are germ line mutations in the RET proto-oncogene. Examination of the mutated codons highlighted a genotype-phenotype correlation between the transforming activity, disease onset and aggressiveness of MTC [2–4], implicating that manifestation and clinical progression is conditioned by the type of mutation. The mutations in RET are leading to a constitutive activity of the receptor tyrosine kinase, resulting in a gain-of-function. This affects downstream signaling networks which modulate apoptosis, cell proliferation and metastasis, whereas functional knockdown of the RET receptor could reverse the effects [2, 5–8]. Characterized by the presence of MTC only or secondary malignancies like pheochromocytoma and parathyroid hyperplasia, this cancer type can be divided into three clinical subtypes, MEN 2A, MEN 2B, and FMTC. Around 25% of MTC occurs as the autosomal dominant inherited form as a component of the multiple endocrine neoplasia type 2 (MEN 2) syndromes [1]. While MEN 2A is the most common syndrome, MEN 2B represents the most aggressive form with high risk of early onset of the disease and development of metastasis [9]. MTC is a slowly progressing tumor, metastasizing to cervical and mediastinal nodal groups and to distant organs such as lungs, liver, and bones in 50 and 20% of cases, respectively [10]. Total thyroidectomy remains the only effective treatment and only prior to metastasis; once cervical lymph nodes are present, the disease becomes residual with little chances of cure. In addition, some patients also develop recurrent disease [11]. Treatment with tyrosine (or multi-) kinase inhibitors (e.g., vandetanib or cabozantinib) for advanced and metastatic MTC shows only moderate effect and still carries high risk of severe adverse events, considering these drugs solely for patients where the benefits outweigh toxicity [12–14].

Recent work in various cancers elucidates the tremendous influence of microRNAs (miRNAs), non-protein-coding, endogenous, small RNAs altering gene expression by translational repression or mRNA cleavage, as tumor suppressors or oncogenes (OncomiRs, MetastamiRs). Due to their impact on several biological pathways in human cancers, miRNAs are broadly discussed as prognostic biomarkers and therapeutic targets [15–17]. Several miRNAs have been shown to be dysregulated in MTC, participating in tumor progression [18–24] and more importantly that some miRNAs, like miR-183, miR-375, and miR-21, strongly correlate with metastasis in MTC patients [18, 23].

MiR-182 is a member of the miR-183 cluster (miR-183, miR-182, miR-96), which reveal important functions and influence on tumor development in several cancers. Interestingly, miR-182 mediates cell motility and migration in medulloblastoma after knockdown of the single members of the miR-183 cluster [25–28]. Furthermore, overexpression of miR-182 demonstrated highly aggressive features, strongly contributing to enhanced invasion, survival and chemoresistance through repression of a plethora of targets e.g., FOXO3, MITF, MTSS1, or PDCD4 [29–32]. The significance of miR-182 is also supported by a study that proposed circulating miR-182 as blood based biomarker for tumor progression [33]. Beyond, in vivo treatment with anti-miR-182 in an orthotopic xenograft model for ovarian cancer could reduce tumor burden via restoration of miR-182 targets [34]. In thyroid cancer, increased mir-182 expression has been reported only in papillary and follicular thyroid carcinomas but not MTC [35, 36].

In this study, we found a significant upregulation of miR-182 in tissues of a cohort of medullary thyroid carcinomas compared to normal thyroid tissue and in cell lines expressing RET mutations. HES1 was identified as a direct target of this miRNA through binding in the 3′untranslated region (3′UTR) and showed a significant downregulation in vitro and in MTC tissues. Analyzing the biological function of the miR-182-HES1 axis revealed the ability to regulate migration and invasion but not proliferation of thyroid cell lines. Moreover, we show that loss of HES1 is correlated with decreased expression levels of Notch1, that implies a possible negative feedback loop mediated through RET induced miR-182 expression. Our data suggest that the expression of miR-182 is involved in malignant progression of medullary thyroid carcinoma, providing new insights in pathogenesis and treatment relevant targets.

## Methods

### Vector construction, production and transduction

The adenoviral (Ad) vectors Ad.RET∆TK and Ad.GFP have been previously described [6]. Multiplicity of infection was chosen to allow 100% transduction of target cells. A 245-bp fragment containing miR-182 was amplified by PCR, subcloned into pJET1.2 (ThermoFisher Scientific) and subsequently inserted into the PmeI and MluI restriction sites of the lentiviral vector plasmid pLemiR. CDS fragment coding for RETM918T was obtained from the pJ7omega vector [2] by HindIII digestion and inserted into the EcoRI-restricted pWPXL after blunting reaction. Lentivector-based miRZIP-182 and miRZIP-scramble plasmids were purchased from System Biosciences. Lentiviral vectors encoding miR-182, miR-scramble, RETM918T, miRZIP-182 and miRZIP-scramble were produced by cotransfection of HEK293T cells using the plasmids pMD2.G and psPAX2 (Addgene) as described [37]. For stable expression of the transgenes, 1×10^5^ NThy-ori 3.1 cells were plated with antibiotic-free medium containing high-titer lentiviral particles and incubated for 72 h. The medium was removed and fresh RPMI supplemented with 2 μg/ml puromycin was added for selection.

### Cell lines and cell culture

NThy-ori 3.1 human primary thyroid epithelial cells derived from normal thyroid tissue of an adult individual were purchased from Sigma-Aldrich and maintained according to the supplier’s instructions. Cells were cultured in RPMI with sodium pyruvate, 2 mM L-glutamine, 100 μg/ml penicillin, 100 U/ml streptomycin, and 1.25 μg/ml amphotericin B. NThy-ori 3.1 stable cell lines expressing RETM918T, miR-182, or miR-scramble were additionally cultured with 2 μg/ml puromycin. TT cells, a human MTC cell line expressing MEN2A type RETC634W, were grown in HAM’s F12 medium supplemented with the above described additives. NThy-ori 3.1 cells were transfected with Turbofect transfection reagent (Thermo Scientific). Plasmids pRFP-C-RS-shHES1, pRFP-C-RS-shControl for HES1 knockdown, and pcDNA3.1-FLAG-HES1-wild-type for HES1 overexpression experiments were kindly provided by P. A. Zweidler-McKay. Empty pcDNA3.1 from Invitrogen™ was used as control vector. Proteasome inhibitor MG-132 (Cell Signaling) was added to the medium 24 h post transfection at a final concentration of 40 μM and cells were incubated for 5 h.

### RNA isolation and qPCR

RNA was extracted using the NucleoSpin RNA Kit (Macherey-Nagel). For qRT-PCR, RNA was reverse transcribed with Omniscript RT Kit (Qiagen). The cDNA samples were mixed with iQ™SYBR Green Supermix and analyzed on iQ5 Multicolor Real-Time PCR Detection System (Bio-Rad). Relative gene expression was calculated by the comparative Ct method using actin or GAPDH for normalization as indicated. PCR primers for detection of the specific DNA products are presented in Additional file 1: Table S1.

### TaqMan PCR analyses

MicroRNAs from tumor tissues and cells were extracted using the NucleoSpin® miRNA Kit from Macherey-Nagel. Concentration and purity of the small RNA fraction was measured with NanoDrop ND-1000 Spectrophotometer (Thermo Fisher Scientific) and 25 ng of small RNA fraction was used for reverse transcription reaction. Conversion of miRNAs to cDNA was carried out with TaqMan® MicroRNA Reverse Transcription Kit (Applied Biosystems). MicroRNA assay was performed using TaqMan Small RNA Assay with 7900HT Fast Real-Time PCR System (Applied Biosystems) following the instructions. Specific microRNA primers used for the detection of hsa-miR-182 and RNU6B were purchased from Applied Biosystems. Relative miRNA expression was calculated by the comparative Ct-method using RNU6B for normalization.

### Western blotting and immunofluorescence

Cells were lysed in RIPA buffer protease inhibitor mixture Complete Mini (Roche) and total protein concentration was quantified by Bradford assay (Bio-Rad). Equal amounts of protein were separated by SDS-PAGE and transferred to nitrocellulose membranes (Amersham Biosciences). Samples were probed with antibodies against RET (C-20), p-RET (Tyr1062), Notch1 (C-20), HES1 (H-140), Deltex1 (D-20) from Santa Cruz, cleaved Notch1 (Val1744) (Cell Signaling), NF-κB p65 and NF-κB p65 (pS536) from BD Biosciences, TATA-binding protein (TBP; Abcam), and β-Actin (Sigma).

For immunofluorescence, cells were grown on glass slides for 24 h and fixed in 4% paraformaldehyde, permeabilized with 0.1% Triton X-100 in PBS, and blocked with 10% FCS and 1% BSA in PBS. Cells were incubated with primary antibody for NOTCH1, HES1, Deltex1 and NF-κB p65 (Santa Cruz) diluted in 3% BSA in PBS/0.02% Triton X-100, washed in PBS, and incubated for 60 min with fluorescence-labelled secondary antibody (Thermo Scientific). Cell nuclei were stained with DAPI solution. Images were obtained using an inverted confocal laser scanning microscope (Zeiss).

### ChIP

Chromatin immunoprecipitation was performed essentially as described [38]. Protein-DNA complexes were immunoprecipitated using anti-NF-κB p65 (BD Biosciences) or control IgG. Primer sequences for the binding sites are listed in Additional file 1: Table S1.

### Cloning of promoter and 3′UTR-constructs and luciferase reporter assay

200 bp of the promoter region of miR-182 harboring the NF-κB p65 binding site and the promotor deletion fragment were amplified from genomic DNA and cloned into KpnI and XhoI restriction sites of the pGL3 basic vector (Promega). For p65 expression, the CDS was amplified from cDNA and inserted into pCDNA3.1/V5-HIS TOPO (ThermoFisher Scientific). The 3′UTR of HES1 mRNA containing the complementary sequences to the miR-182 seed sequence was amplified from genomic DNA and subcloned into pcDNA3.1. After digestion, the 3′UTR fragment was inserted downstream of the luciferase reporter of pMIR-REPORT vector (Ambion/Invitrogen). Mutation of DNA sequences was carried out with QuikChange XL Site-Directed Mutagenesis Kit (Stratagene). Reporter activity was assayed 48 h post-transfection using the Luciferase Reporter Assay System (Promega). Samples were normalized to total protein concentration in the cell extract. For primer sequences see Additional file 1: Table S1.

### Matrigel invasion assay

Cell invasiveness was assessed as described [39]. Briefly, 2×10^5^ cells in 1.5 ml of serum-free DMEM were added to the wells of 8 μm pore membrane Boyden chambers coated with 3.1 mg/ml diluted Matrigel basement membrane matrix (BD Biosciences). Medium containing 20% FCS was added as chemoattractant in the lower chamber and cells were allowed to invade for 36 h. After removing of cells that had not penetrated the filters by scrubbing with cotton swabs, they were stained with DAPI for five minutes and at least five sections per membrane were photographed. Labelled cells were counted from images with ImageJ software (https://imagej.nih.gov/ij/).

### Scratch assay

Cells were grown in monolayers and wounded with a pipette tip. Detached cells were washed off with PBS and new medium was added. Migration of cells was measured as reduction of the wound area in each photographed field. At least five fields were photographed for each condition each time and the gap was calculated using ImageJ software.

### Proliferation and XTT assay

For XTT assay, cells seeded in 24-well plates were incubated with TACS XTT labeling mixture (Trevigene). After 2 h supernatant of the cells were pipetted into 96-well plates and the conversion of XTT to formazan was quantified by measuring the spectral absorbance at 490 nm. Cell quantity was measured by seeding 2x10^4^ cells in 6 cm^2^ plates and counted every 24 h via trypan blue exclusion.

### Clinical samples

MTC samples from patients with RETM918T (*n* = 8), RETC634Y (*n* = 2), RETC620R (*n* = 2), and RETC618S (*n* = 1) and tissues from normal thyroid gland (healthy donor, HD, *n* = 6) obtained under a protocol approving the patients’informed consent were investigated by real-time RT-PCR. Snap frozen material was ground in liquid nitrogen.

### Statistical analysis

All experiments were carried out in triplicate. The quantitative values were expressed as mean ± standard deviation (SD) and the hypothesis test for significance between two groups utilized the Student’s *t*-test. On MTC patient samples two-tailed Mann-Whitney test was performed using GraphPad Prism version 5.01 for windows, GraphPad software, San Diego California USA, www.graphpad.com.

## Results

### Upregulated miR-182 in hereditary MTC promotes invasion and migration in vitro

**Fig. 1:**
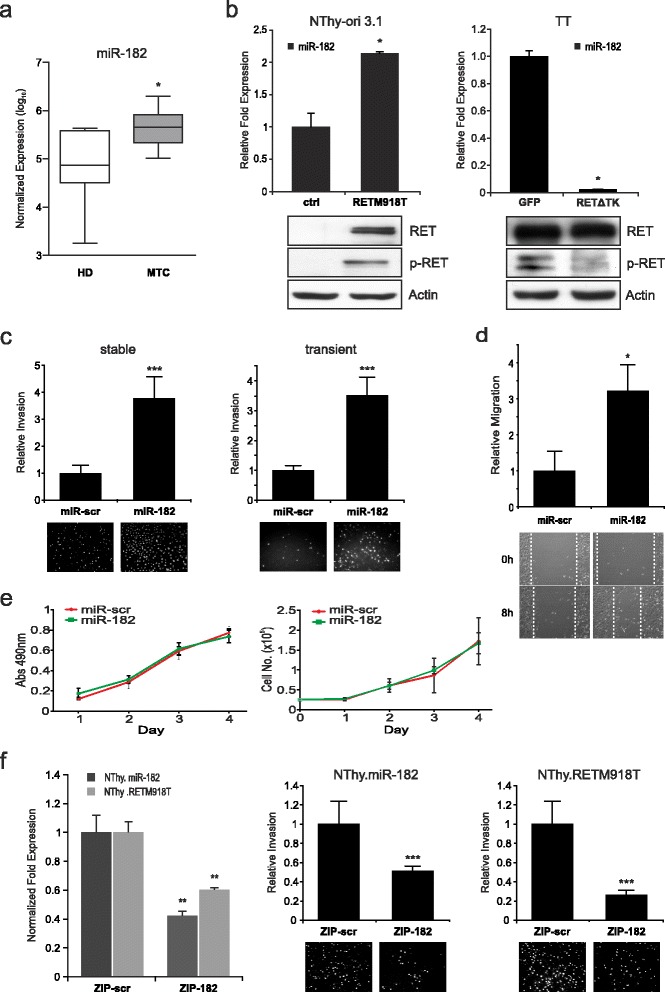
Identification of miR-182 in MTC and effect of miR-182 on cell migration and invasion in vitro. **a** TaqMan single assays for miR-182 expression in patient samples. Expression values are normalized to RNU6B and shown in log10 scale. Mann-Whitney test was used for statistical analyses. Values are means ± SD. HD healthy donor, MTC medullary thyroid carcinoma. **b** Analysis of miR-182 transcripts in NThy-ori 3.1 cells harboring the RETM918T mutation and TT cells transduced with Ad.RET∆TK (RET∆TK) and control Ad.GFP (GFP). For quantification values are normalized to RNU6B and the controls are set as one. Student’s *t*-test was used for statistical analysis. Presence of RET expression was confirmed in immunoblots using antibodies against phosphorylated RET (p-RET) and RET. Actin served as loading control. **c** Invasion of NThy-ori 3.1 cells with stable (*left*) and transient (*right*) expression of miR-182 or miR-scramble control (miR-scr). After 36 h migrated cells were DAPI stained and documented with fluorescence microscope. Fold changes of invasion were calculated relative to the control (set as 1). Representative images are shown from individual experiments for miR-scr and miR-182 below graphs. **d** Wound closure assays of NThy.miR-182 cells with representative images at 0 and 8 h after scratching (*bottom*). *Bar graphs* indicate quantitative data of three independent experiments after 8 h. Fold changes are relative to the controls. Values are means ± SD. **e** Proliferation and viability assays on NThy-ori 3.1 cells expressing miR-182 or miR-scr control. Specific amounts of cells were seeded and either counted every 24 h for 4 days (*right*) or treated with XTT for 1 h and absorbance was measured at 490 nm (*left*). Experiments were repeated three times and Student’s *t*-test was performed on quantification. **f** Inhibition of miR-182 through miRZIP-182 (ZIP-182) reduces migration. MiR-182 knockdown was analyzed by TaqMan qRT-PCR in stable NThy.miR-182 and NThy.RETM918T cell lines. MirZIP-scramble (ZIP-scr) was used as control. Relative expression levels were calculated with 2^-∆∆Ct^-method and normalized to RNU6B (*left panel*). Invading cells seeded on Matrigel coated transwell chambers were counted 24 h post transfection with miRZIP-182 or miRZIP-scramble (*right panels*). After 36 h migrated cells were DAPI stained and analyzed. Graphs show three individual experiments with representative images. Statistical significance is indicated as * *p* < 0.05, ** *p* < 0.01, *** *p* < 0.001

In a first attempt, we quantified miR-182 expression by TaqMan qRT-PCR in a cohort of patient samples harboring the highly aggressive RETM918T mutation. Our analysis revealed that miR-182 is expressed at significantly higher levels in MTC compared to healthy donor tissue (Fig. 1a; MTC *n* = 13, HD *n* = 6). To further elucidate the role of oncogenic RET in the activation of miR-182, we generated a stable NThy-ori 3.1 thyroid cell line derivative expressing RETM918T (NThy.RETM918T). In these cells, the expression level of miR-182 was significantly higher than in parental control cells (Fig. 1b, left). Additionally, intrinsic oncogenic RET autophosphorylation, which maintains their neoplastic properties, was functionally disrupted in TTs, a MTC-derived cell line harboring a RETC634W mutation, by a dominant-negative truncated form of RET, termed RET∆TK [5]. Ectopic expression of RET∆TK abolished phosphorylation of the RET protein and led to the loss of miR-182 expression (Fig. 1b, right), demonstrating that endogenous oncogenic RET signaling is involved in the regulation of miR-182 in MTC cells. To assess the oncogenic effect of altered miR-182 levels and its potential influence on the development of the aggressive behavior of MTC, we investigated NThy-ori 3.1 cells genetically modified to stably express miR-182 (NThy.miR-182) for their invasive capacity. MiR-182 considerably enhanced the number of invading cells compared to control cells in transwell Matrigel assays. Likewise, a higher invasiveness was also apparent in NThy-ori 3.1 cells with transient miR-182 expression (Fig. 1c). Moreover, cells with ectopic expression of this miRNA are more capable in closing an artificial wound scratched into a confluent monolayer (Fig. 1d). To demonstrate that their augmented invasive and migratory behavior is not a cause of cell proliferation, we compared the growth rates of miR-182 and miR-scramble control (miR-scr) transduced cells. Neither cell counting nor XTT assay showed a significant difference between both cell populations (Fig. 1e). In contrast to the increased invasive capability observed upon upregulation of miR-182, introduction of miRZIP-182 antisense miR into NThy.miR-182 and NThy.RETM918T expressing cells, notably reducing the miR-182 level (Fig. 1f, left), significantly impaired their invasiveness (Fig. 1f, middle and right panel). Considering that RETM918T is the highest risk mutation with strong migratory abilities [2], and is also the most common mutation detected in 57% (8/14) of patients in the pN1p/cM1 MTC stage [40], these results support the role of miR-182 as a contributor of RET oncogene-induced metastatic development.

### MiR-182 represses HES1 through direct binding at the 3′UTR

**Fig. 2:**
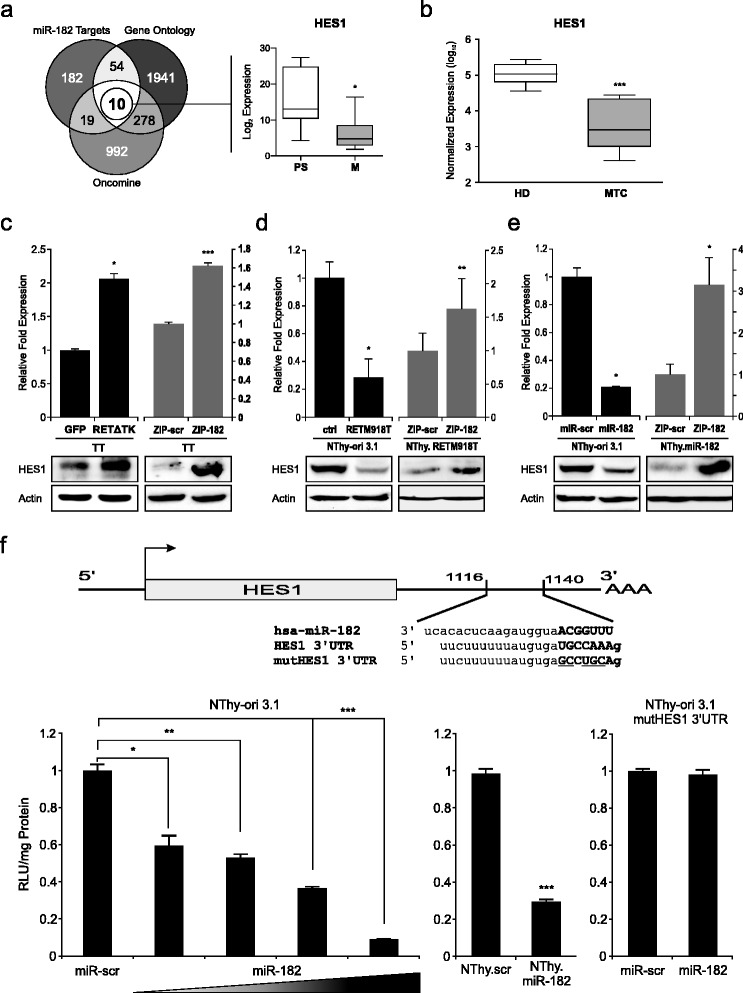
Identification of HES1 as a target of miR-182 in MTC. **a** Schematic overview of *in silico* analyses for identification of potential miR-182 targets. Venn diagram displays number of genes from different sources narrowed down through cross comparison. *Left* circle represents a cumulative list of miR-182 targets from three different prediction algorithms (TargetScan, PicTar, DIANA Lab). *Right* circle shows the amount of genes related to cancer according to gene ontology terms. *Bottom* circle depicts a list of differentially regulated genes in MTC received from the Oncomine database. A set of ten targets was identified and their expression examined preliminary from Oncomine obtained data comparing primary (PS) *versus* metastatic (M) MTC tissues displaying a significant downregulation of HES1 (*right graph*). **b** Validation of HES1 expression in MTC samples compared to healthy donor tissue. Values are normalized to RNU6B. Mann-Whitney test was used for statistical calculation. **c-e** HES1 expression values with activated RET and miR-182 status in different cell line models. TT cells infected with Ad.RET∆TK and control Ad.GFP (**c**, *left*) or transfected with miRZIP-182 and control miRZIP-scramble (**c**, *right*), RETM918T (**d**, *left*) and miR-182 (**e**, *left*) or miR-scr control transfected NThy-ori 3.1 cells, as well as NThy.RETM918T (**d**, *right*) and NThy.miR-182 cells (**e**, *right*) transfected with miRZIP-182 or miRZIP-scramble were harvested 24 h after treatment and analyzed for HES1 expression on RNA and protein level. For qRT-PCR values were normalized to actin and compared relative to the controls Ad.GFP (**c**) or ZIP-scr (**d-e**). Statistical significance was calculated by Student’s *t*-test. Actin served as loading control in immunoblots. **f** Scheme of the HES1 3′UTR with a putative miR-182 binding site and the mutated sequence (*top*). Luciferase activities were measured 24 h after cotransfection of NThy-ori 3.1 cells with HES1 3′UTR and increasing amounts (0.5, 1.0, 2.0 μg) of miR-182 or mutHES1 3′UTR (*right*) and 1 μg of miR-182 or miR-scr (*left*). Stable miR-182 NThy-ori 3.1 were transfected with HES1 3′UTR as positive control (*center*). Results are shown as fold increase relative to scramble control which was set as 1; * *p* < 0.05, ** *p* < 0.01, *** *p* < 0.001

To identify molecular mechanisms by which miR-182 contributes to tumor progression of MTC, we performed bioinformatic analyses using several computational algorithms, including TargetScan, PicTar, and DIANA Lab to search for target genes of this miRNA. The predicted list of potential targets was narrowed down by combining data from Gene Ontology and the Oncomine database comprised of differentially expressed genes in medullary thyroid carcinoma and eventually led to ten putative targets. In a first step, the expression status of these ten genes was analyzed in the Oncomine database pointing at HES1 as a promising candidate that is clearly reduced in metastatic versus primary MTC tissues (Fig. 2a, right). A significant downregulation of HES1 in thyroid tumors with high miR-182 level compared to patient samples from normal thyroid glands (Fig. 1a), was also verified by qRT-PCR (Fig. 2b). To determine whether HES1 expression is related to endogenous RET activity, we examined HES1 expression in TT cells in the absence and presence of the RET inhibitor ∆TK. As shown in Fig. 2c, HES1 expression is elevated in RET∆TK transduced cells compared to the control in both, on RNA and protein level. The same effect on HES1 could be obtained after inhibition of miR-182 in these cells. (Fig. 2c, right). Vice versa, NThy-ori 3.1 cells carrying active RETM918T showed a markedly decreased HES1 expression (Fig. 2d, left panel), which is also visible in response to miR-182 overexpression in parental NThy-ori 3.1 cells (Fig. 2e, left panel). In line with the above data, HES1 expression was recovered upon knockdown of miR-182 in NThy-ori 3.1 cells stably expressing either RETM918T or miR-182 (Fig. 2d and e, right panels). Taken together, the HES1 gene is regulated through miR-182 in response to intrinsic oncogenic RETM918T signaling.

To determine if miR-182 directly targets HES1, we cloned the HES1 3′UTR bearing the putative miRNA binding site into the pMIR-REPORT plasmid (Fig. 2f). Reporter assays using the miRNA-3′UTR of the HES1 gene in NThy-ori 3.1 cells showed a strong reduction of luciferase activity when cotransfected with increasing concentrations of miR-182 expression plasmid (Fig. 2f, left). This miR-182-dependent inhibition of the reporter construct was also seen in NThy-ori 3.1 cells that stably express miR-182 (center panel), whereas miR-scramble control had no effect in both cell lines. In contrast, mutation of the miR-182 seed sequence in the HES1-3′UTR did not lead to any changes in luciferase activity (right panel), which strongly supports the direct interaction of miR-182 with the HES1-3′UTR via this sequence. In summary, these results provide evidence that a repression of HES1 can be induced through oncogenic RET by directly binding of miR-182 at the 3′UTR of HES1.

### Loss of HES1 contributes to the aggressive phenotype of MTC and negatively effects the Notch1 signaling pathway

**Fig. 3:**
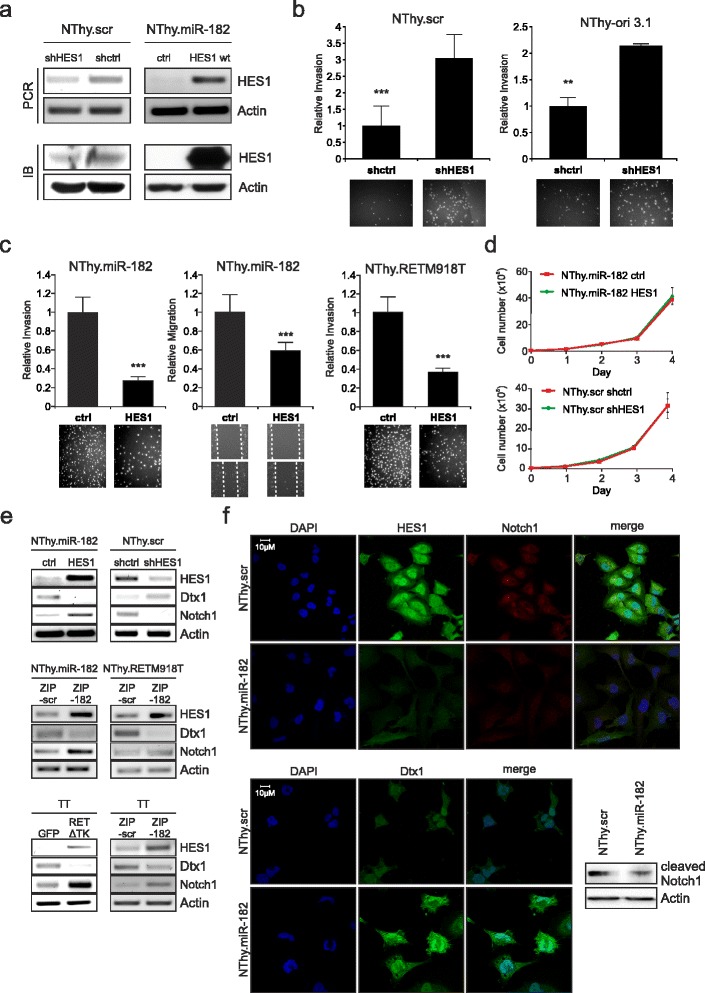
HES1 status alters invasive behavior in thyroid cells and affects expression of Notch1 and Dtx1. **a** Validation of HES1 knockdown in stable NThy.scr and HES1 overexpression in NThy.mir-182 by shHES1 and HES1 wild type (HES1 wt), respectively, on mRNA and protein level. Empty pcDNA3.1 and shControl (shctrl) plasmids were used as controls. Actin shows equal loading of protein. **b-c** Invasion and migration of thyroid cells after knockdown of HES1 (**b**) or HES1 overexpression (**c**). Values are relative fold compared to related controls of three individual experiments with representative images from fluorescence microscopy for Boyden chamber and bright field microscopy for wound healing assays; ** *p* < 0.01, *** *p* < 0.001. **d** NThy.miR-182 (*top panel*) and NThy.scr cells (*bottom*) were transfected with plasmids encoding HES1 wild type or shHES1. After one day specific amounts of cells were seeded and counted every 24 h to determine proliferation. **e** Detection of Notch1, Dtx1, and HES1 transcript levels in indicated thyroid cell lines after specific treatment: HES1 up- and downregulation (*top*), depletion of miR-182 (*center*, *bottom right*) and blockade of RET-associated signaling (*bottom left*). Cells were transfected with corresponding control plasmids and RNA was isolated 24 h later. Actin was used for equal loading. **f** Immunofluorescence staining of HES1, Notch1, and Dtx1 in miR-182 expressing NThy-ori 3.1 cells compared to control cells with miR-scr. Nuclei were counter-stained with DAPI and fluorescence was visualized by confocal laser scanning microscopy. Immunoblot indicates cleaved Notch1 and actin in NThy.miR-182 cells (*bottom*, *right*)

HES1 is a negative transcriptional regulator mainly involved in neuronal development and a downstream effector in the Notch signaling pathway [41, 42]. To clarify the role of HES1 in the progression of RET-associated cancer, we modified its expression level in NThy-ori 3.1 cell lines using plasmids containing the coding sequence of wild type HES1 or shHES1 for optimal overexpression and gene-specific knockdown as shown in Fig. 3a. Compared to cells treated with scramble-shRNA, depletion of endogenous HES1 in NThy.scr as well as parental NThy-ori 3.1 led to an accelerated invasion rate (Fig. 3b). The opposite effect was observed when HES1 was ectopically expressed in invasively growing NThy-ori 3.1 cells with stable miR-182 or mutant RETM918T expression level. HES1 also inhibits the faster wound healing of miR-182 expressing cells. The results indicated that overexpression of HES1 prevents thyroid cells from moving and is associated with a marked decrease of their invasive capacity (Fig. 3c). In contrast, we could not find any significant changes in cell viability or proliferation after altering the HES1 level in these cell lines (Fig. 3d).

HES1 is a member of the Notch pathway and a direct target of Notch1. It has been shown that Notch1 is not expressed in MTC, thus leading to impaired signaling including loss of HES1 expression. In addition, overexpression of Notch1 had tumor suppressive effects in MTC [43, 44]. Notch1 can be regulated through HES1 and Deltex1 (Dtx1) in a negative feedback loop, in which HES1 is able to bind to the Dtx1 promoter repressing its transcription which, in turn, recovers Notch1 expression [45]. In a first step, we analyzed the transcript levels of Notch1, Dtx1 and HES1 in our cell culture models. As shown in the upper panel of Fig. 3e, restoration of HES1 expression in NThy.miR-182 cells caused a significant increase of Notch1 and depletion of Dtx1. Conversely, Dtx1 was upregulated by shRNA-mediated repression of HES1 in NThy.scr cells, while Notch1 decreased. Moreover, we demonstrate that HES1 and Notch1 expression is rescued through silencing of mir-182 in miRZIP-182 transfected NThy.miR-182 cells. The observed changes are accompanied by the loss of the Dtx1 transcript (Fig. 3e, center). A similar result was found in TT cells, where inhibition of miR-182 increased the HES1 and Notch1 levels, whereas Dtx1 transcripts were lower compared to the control. It is also noteworthy that functional knockdown of oncogenic RET activity mediated through the RET∆TK mutant provided the same changes in Notch1, HES1, and Dtx1 expression as seen in miR-182 depleted TT cells (Fig. 3e, lower panels). Immunofluorescence experiments revealed strong signals for HES1 and Notch1 in the NThy.scr control, which show particularly high concentrations in the nucleus compared to cells with miR-182 that have high levels of Dtx1 (Fig. 3f). Since only cleaved Notch1 is capable of entering the nucleus to turn on transcription of target genes, nuclear staining is indicative for active Notch1. Specifically, a reduction of cleaved Notch1 in miR-182 expressing cells is also evident from Western blot (Fig. 3f, bottom right panel). Interestingly, MG-132 treatment could rescue cleaved Notch1 and HES1 expression, not only reverting the effect of miR-182 upregulation but also counteracting HES1 knockdown (Additional file 2: Figure S1). This implies that miR-182-mediated blocking of HES1 expression is responsible for the activation of metastatic traits in RET oncogene-dependent cancers, since loss of this transcription factor leads to a higher invasive potential of the cells. Moreover, the correlation with Notch1 and Dtx1 also indicates that HES1 ablation has a negative impact on the Notch signaling pathway.

### Oncogenic RET-activated NF-κB regulates miR-182

**Fig. 4:**
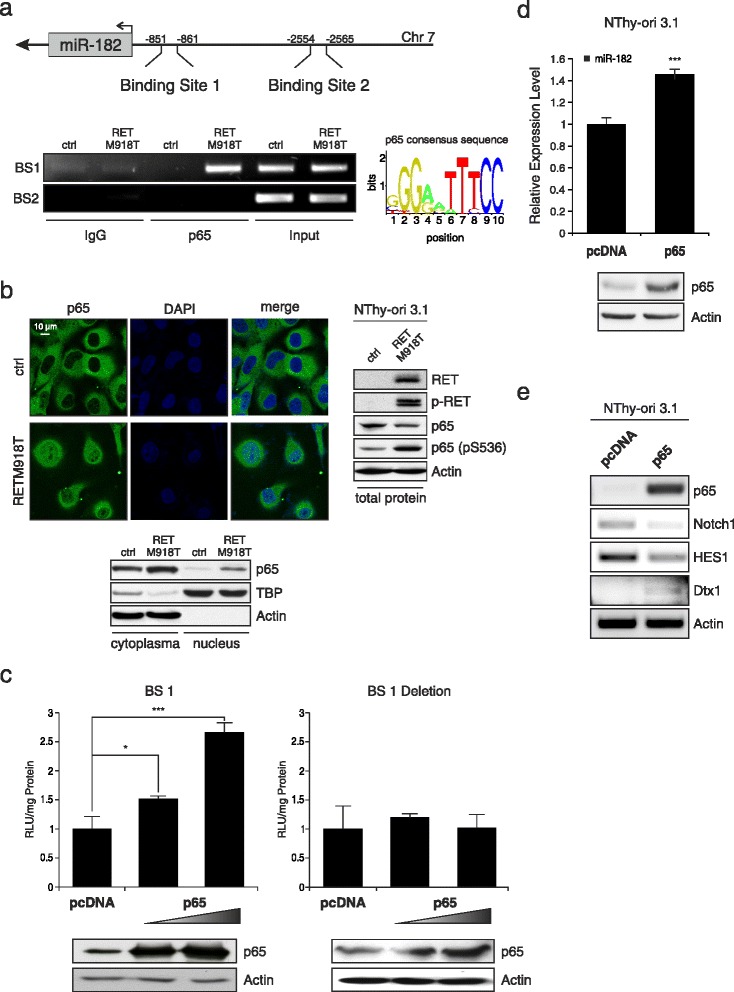
Oncogenic RET-activated NF-κB induces miR-182 expression. **a** Schematic diagram of the promoter region with two putative p65 binding motifs at positions −851 (binding site 1, BS1) and −2554 (binding site 2, BS2) upstream of the transcriptional start of miR-182 (*top*). The graph shows the consensus sequence used for identification of p65 binding sites. ChIP assays were performed using anti-NF-κB p65 and IgG as negative control (*bottom*). **b** Localization and expression of NF-κB p65 in thyroid cells stably expressing mutant RETM918T analyzed by immunofluorescence and Western blot. Blots were probed with RET, p-RET, NF-κB p65, NF-κB p65 (pS536), and Actin, or TBP for equal loading of the cytoplasmic and nuclear fraction, respectively. Western blot on the right represents total protein lysate. DAPI was used for nuclear staining. **c** Luciferase assays of NThy-ori 3.1 cells cotransfected with 1 μg miR-182 promoter (*left*) or BS1 deletion construct (*right*) and increasing amounts (1.0, 2.0 μg) of p65 expression plasmid or pcDNA (1 μg) as control. Reporter activities are shown as fold activation relative to the control after normalization to total protein concentration in cell extracts at 24 h post transfection. Bar graphs are means ± S.D; * *p* < 0.05, *** *p* < 0.001. Expression levels of NF-κB p65 and actin as loading control are indicated by Western blot. **d** Expression of miR-182 in NThy-ori 3.1 cells transfected with p65 or pcDNA was determined by TaqMan qRT-PCR. For relative expression level empty pcDNA was set as one; *** *p* < 0.001. **e** Effect of ectopic NF-κB p65 expression in normal thyroid cells on Notch1, HES1, and Dtx1 RNA levels. Actin served as loading control

The question that then remained to be answered was how miR-182 is connected to activated RET signaling and tumor aggressiveness. Mutations of the RET proto-oncogene enable the kinase receptor to activate a plethora of signaling pathways related to cancer. We first conducted an *in silico* screening of the 4 kb region upstream of the miR-182 transcription start using the JASPAR Database. We found several predicted binding sites of different transcription factors including two responsive sequence elements of NF-κB p65. In previous studies it has been reported that oncogenic RET induces NF-κB-dependent transcriptional activity in TT cells via RAS-RAF-IKKβ [46] and that it is frequently activated in MTC [47]. As shown in Fig. 4a, we determined the two conserved putative p65 motifs, named BS1 and BS2 starting proximal from the miR-182 transcription site, which were verified by ChIP assays in NThy-ori 3.1 cells harboring constitutively activated RETM918T. Endogenous DNA-protein complexes were immunoprecipitated with a specific antibody against NF-κB p65 and IgG as a negative control. Subsequent PCR analysis demonstrated a strong binding of p65 to the BS1 element, while there is no enrichment at the more distant second binding site in the miR-182 promoter.

Moreover, immunofluorescence staining and Western blot analysis of fractionated protein lysates revealed that stable expression of mutated RET in NThy-ori 3.1 promotes p65 activation by enhancing its translocation to the nucleus (Fig. 4b, left). Additionally, phosphorylated RET is accompanied by phosphorylation of p65 (Fig. 4b, right), which is also necessary for transcriptionally active NF-κB [48, 49]. In order to determine responsiveness of the miR-182 gene to NF-κB activity, we generated luciferase reporter constructs containing the promoter sequence with the BS1 motif and cotransfected NThy-ori 3.1 cells with a p65 expression plasmid. Promoter upregulation in response to ectopic p65 occurred in a dose-dependent manner reaching a 2.5-fold increase of luciferase activity after 24 h compared to empty control vector (Fig. 4c). Consistent with our prediction, a complete loss of reporter activity was observed when the BS1 site was deleted. In line with this, enforced expression of p65 in normal thyroid cells caused a moderate but significant increase of miR-182 (Fig. 4d), leading to reversed effects on the expression of Dtx1, HES1, and Notch1 than seen in NThy.RETM918T or TT MTC cell lines in response to miRZIP-182 treatment (Fig. 3e) with a downregulation of HES1 and Notch1 and increased Dtx1 transcript levels (Fig. 4e). These data indicate that RET-activated NF-κB p65 directly binds to and transactivates miR-182, thereby repressing the Notch tumor suppressor signaling pathway.

## Discussion

Understanding the differential development and disease progression of patients with MTC and MEN2-syndromes caused by specific RET mutations, there remain critical points for an effective diagnostic and therapeutic management. Molecular testing, biochemical analysis, and the well-established genotype-phenotype correlations for MEN2 patients provide information about signaling processes by which RET mutations cause tumors, yet, exact mechanisms of specific oncogenic pathways are not fully understood especially for advanced stage MTC and metastasis. Hence, we investigated the relevance of RET‐induced miRNAs for its oncogenic activity in cancer progression. We could show that miR‐182 is upregulated in MTCs harboring mutated RET in codons 918 and 634 and demonstrated that enforced expression of miR‐182 is necessary and sufficient to promote an aggressive phenotype. This process is induced by highly oncogenic RET via activation of NF-κB, which directly binds in the upstream region of miR-182. Increased miR‐182 represses HES1, a key player in the Notch signaling pathway that also acts as a tumor suppressor in MTC. Interestingly, loss of HES1 expression leads through a negative regulatory loop to the reduction of Notch1, thus giving an explanation for the lost Notch pathway in MTC.

So far, only a few miRNA profiling studies of medullary thyroid carcinomas have been published [18–20] and although limited data are available on a potential role in MTC [18, 19, 23, 50], compelling evidences reveals their influence in endocrine tumor development. Mir-182 belongs to the miR-183 ~ 96 ~ 182 cluster, of which one member, miR-183, was found to be upregulated in MTC and associated with metastasis, mortality, and residual disease [18, 19]. MiR-182 is overexpressed in several cancer types and involved in epithelial-mesenchymal transition (EMT), migration, and drug resistance, and thereby seems to have a strong impact on the metastatic potential of tumor cells [27, 29, 31, 51]. Inhibition of miR-182 in a xenograft model for ovarian cancer could drastically reduce tumor size and metastatic spread, implicating this miRNA not only as prognostic marker but also as therapeutic target [34]. As recently reported, miR-182 is also upregulated in papillary thyroid cancer and controlling cell proliferation and invasion [36]. In our study, functional analyses on miR-182 clearly revealed tumorigenic actions that are characterized by the stimulation of migratory and invasive properties in non-migrating thyroid cells. Its function in promoting aggressive traits in RET-dependent cancer is reinforced by the decrease of motility and invasion after miR-182 depletion in RETM918T transformed cell lines. Since reduced miRNA expression is also seen in response to functional knockdown of RET in TT cells, this provides evidence that miR-182 is equally activated in RETC634-mutated MTC. Uncovery of the specific role of this miRNA in the acquisition of the highly malignant phenotype of MTC and the underlying mechanisms will most likely yield potential therapeutic targets.

**Fig. 5:**
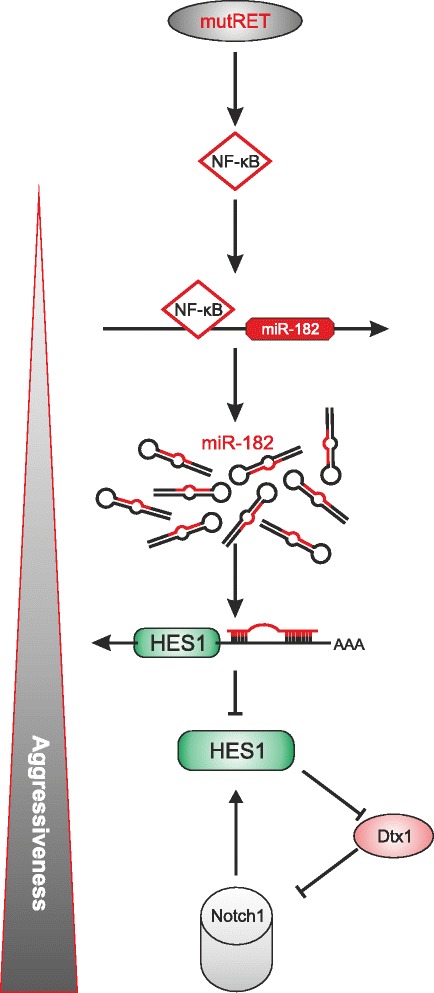
Schematic illustration of the RET oncogene-induced NF-κB-miR-182-HES1 axis that promotes cancer aggressiveness via inhibition of Notch signaling. Mutated RET activates NF-κB which induces miR-182 expression in the nucleus. Enhanced miR-182 represses the negative transcriptional regulator HES1 through binding to its 3′UTR. The inhibitory influence of HES1 on Dtx1, a suppressor of Notch, is attenuated with the consecutive effect of reducing Notch1 level in the cells. As consequence, Notch1 cannot exhibit its tumor suppressor function in MTC, leading to a more malignant phenotype

We newly uncovered HES1 as an important target of miR-182. The transcriptional inhibitor HES1 is a member of the basic helix-loop-helix family, known to regulate many physiological processes like cellular differentiation, cell cycle arrest, apoptosis and self-renewal, and according to most recent studies, also functions in EMT induction, metastasis, and maintenance of cancer stem cells [52–54]. Depending on the tumor type, HES1 can either act as oncogene or tumor suppressor. In colorectal cancer, where elevated HES1 expression is correlated with distal metastasis and poor prognosis, this protein enables upregulation of EMT markers like vimentin and N-cadherin, thereby promoting the invasive ability of cancer cells [53]. In contrast, downregulation of HES1 in bladder cancer enhanced vimentin and reduced E-cadherin levels, which triggers an EMT phenotype and malignant progression [55]. In accordance, our results show that loss of HES1 through miR-182 causes a strong increase of the migratory capacity in the normal thyroid cells, whereas restoration of HES1 is associated with a lower invasion rate of mutant RET and miR-182 expressing cells. This constellation clearly supports the tumor suppressor function of HES1 in MTC. In this regard, it is of particular interest that HES1 is a direct target of Notch1 and the Notch1 signaling pathway is characteristically absent in MTC. Although in many cancer types Notch1 is an important tumor promoting oncogene, it was shown to possess a tumor suppressive effect in some hematopoietic cancers and especially in neuroendocrine tumors such as MTC [43, 56, 57]. In fact, Notch1 activation led to repression of neuroendocrine markers apoptosis induction and reduced tumor growth making it an interesting molecule for MTC therapy [43, 44, 58–60]. We observed an opposing expression of HES1/Notch1 compared to Dtx1. According to Zhang and colleagues [45], HES1 can directly downregulate Dtx1, which is acting as a negative regulator of Notch1 signaling, through degradation of the intracellular domain of Notch1 in osteosarcoma. These results and our observation, raise the idea that miR-182-mediated repression of HES1 in MTC leads to the loss of the tumor suppressive Notch1 signaling via a potential negative feedback loop with Dtx1, thereby enhancing the aggressive phenotype (Fig. 5).

Little is known about the activation of miR-182 or transcription factors that are involved in its regulation. In glioblastomas, a particularly aggressive form of brain tumor in which miR-182 acts as a tumor suppressor, miRNA expression is dependent on wild type p53. In line with this, several functional p53 binding sites were detected in the promoter region of miR-182 as well as the entire miR-183 ~ 96 ~ 182 cluster resulting in a p53-mediated negative regulation of miR-182 [61]. In addition, other transcription factors like STAT5, Sp1, and β-Catenin that potentially contribute to cancer progression signaling have been reported to bind at the promoter region of miR-182 [62–65]. In our study, we identified an active binding site of NF-κB in the miR-182 promoter that enables transcriptional activation of this miRNA after nuclear translocation in NThy-ori 3.1 stably expressing RETM918T. Several human cancers have constitutively active NF-κB due to the inflammatory microenvironment and various oncogenic mutations. Previous reports indicated that NF-κB is involved in multiple cancer-related processes by either promoting proliferation or suppressing apoptosis and stimulating EMT and angiogenesis, which facilitates distant metastasis [66] Our results suggest that the metastatic influence of NF-κB is mediated via the invasion enhancing abilities of miR-182, whereas in contrast, proliferation is not effected. In case of MTC, it has been shown that NF-κB activity is induced in TT cells harboring a codon 634 mutation depending on IKK-mediated IκBα degradation and functional RAS/RAF/MEKK1 which blocks apoptosis [46]. Accordingly, microarray analysis of MTC patient samples revealed differential expression of genes from tumor progression-related signaling pathways including NF-κB that are associated with the RETM918T mutation [67]. Elevated levels and transcriptional activity of NF-κB have also been described in FTC, ATC, and PTC in which the transcription factor primarily contributes to apoptosis inhibition [68]. Moreover, in PTC BRAFV600E - activated NF-κB signaling leads to the acquisition of apoptotic resistance but also induces expression of matrix metalloproteinase members and promotes thyroid cancer cell invasion [69]. Regarding miRNA regulation, NF-κB exerts a complex network of context-dependent direct and indirect effects. MiR-146, for instance, is a transactivational target of NF-κB that negatively regulates IRAK1 and TRAF6, constituting a negative feedback loop [70]. While in ATC NF-κB was found to upregulate miR-146a altering drug-induced apoptosis [71], ectopic expression of miR-146a/b in breast cancer cell lines reduced NF-κB activity and impaired the cells’ invasive capabilities [72]. Interaction of miR-182 and NF-κB was observed in gliomas, where TGF-β stimulated miR-182 expression abrogates the inhibitory effect of its target cylindromatosis on NF-κB activation, resulting in their enhanced invasion and angiogenesis [73]. The importance of the NF-κB/miR-182 axis in tumor aggressiveness is underscored by Chang et al*.* [74], who observed a strong impact of NF-κB-driven miR-182 on stemness and metastasis of non-small cell lung cancer cells. In contrast to their work, we could not detect any NF-κB binding at the sequence reported, but instead we identified a new active binding site close to the transcription start of miR-182. Further, multiple cross talk mechanisms between Notch and NF-κB have been discussed in connection with cancer development [75, 76]. Our data provide first experimental evidence for miR-182 as new mediator of a cross talk between these two cell fate determining pathways, in which active NF-κB can negatively regulate Notch signaling via miR-182-induced downregulation of HES1.

## Conclusion

In summary, our results demonstrate that miR-182 is activated by highly aggressive RET mutations M918T and C634W in an NF-κB-dependent manner. Of particular importance is that miR-182 reveals its oncogenic capacity in medullary thyroid carcinoma by directly contributing to the invasive behavior through loss of the tumor suppressive HES1/Notch1 signaling circuit. The uncovered regulation between RET, miR-182 and HES1 clearly points toward new prognostic and therapeutic options by using components of the RET-NF-κB/miR-182-HES1/Notch1 axis as potential key targets to restrict cancer progression. Considering the roles of RET in thyroid and lung adenocarcinoma, as well as its potential contributions to regional spread and metastasis in other cancer types [4], our study raises perspectives for therapeutic intervention in multiple aggressive cancer diseases.

## Additional files

Additional file 1:
**Table S1.** Primers used for amplification and Quantitative Real-Time PCR (qRT-PCR). (DOCX 18 kb)
Additional file 2:
**Figure S1.** Inhibition of ubiquitin-dependent degradation of intracellular active Notch1. **a-b** Effect of inhibitor MG-132 on cleaved Notch1 and HES1 expression inNThy.miR-182 (a), NThy-ori 3.1 and NThy.scr transfected with shHES1 or control (b). Cells were treated with 40 μM MG-132 or equal amount of DMSO for 5 h and at 24 h post transfection. Expression of cleaved Notch1 and HES1 were analyzed by WB. Actin was used as loading control. (PDF 3001 kb)

## Abbreviations

EMT: Epithelial to mesenchymal transition
FMTC: Familial medullary thyroid cancer
MEN: Multiple endocrine neoplasia
miR: miRNA, microRNA
MTC: Medullary thyroid carcinoma
qRT-PCR: Quantitative real-time PCR
