# Supplementary material for: MiR-182 promotes cancer invasion by linking RET oncogene activated NF-κB to loss of the HES1/Notch1 regulatory circuit
Source: Mol Cancer. 2017 Jan 26;16:24. doi: 10.1186/s12943-016-0563-x (PMC5267421; doi:10.1186/s12943-016-0563-x)
Supplement: Additional file 1: — Table S1. Primers used for amplification and Quantitative Real-Time PCR (qRT-PCR). (DOCX 18 kb) [file 12943_2016_563_MOESM1_ESM.docx]

**Table S1** Primers used for amplification and Quantitative Real-Time PCR (qRT-PCR)

| **Primer** | **Sequence 5’ 🡪 3’** |
| --- | --- |
| Prom182-BS1-f | CTT GAG ATG CCT TCA GGA CAC |
| Prom182-BS1-r | TCT CCC ATC CAG ACG GCA C |
| Prom182-BS2-f | ACT TCT GCC CTG GTG AGG TG |
| Prom182-BS2-r | GCT CTA GAA CCG GTC ACT GC |
| DelProm182-f | ATT GGT ACC GAA TCA GAT CGC AGC CAG CC |
| DelProm182-r | GAT CTC GAG TCT CCC ATC CAG ACG GCA C |
| HES1-f | AGA TAG CTC GCG GCA TTC C |
| HES1-r | ACC TCG TTC ATG CAC TCG C |
| HES1-UTR-f | AGGCGATCGCACT CCA TGT GGA GGC CGT G |
| HES1-UTR-r | AGCTCGAGTTC TCA AAT AAA CTT CCC CAA AG |
| mutHES1-UTR-f | CACGAGATTTCTTTTTTATGTGAGCCTGCAGATGTTTGAAAATGCTCT |
| mutHES1-UTR-r | AGAGCATTTTCAAACATCTGCAGGCTCACATAAAAAAGAAATCTCGTG |
| Dtx1-f | TAC ATC ATC GAC CTG CAG TCC |
| Dtx1-r | AGA TGT CCA TAT CGT AGG CCG |
| Notch1-f | AGT GCG ACT GTG ACC CTG G |
| Notch1-r | GAC ATG GGT TGG ACG CAC AC |
| p65-f | CAT ATG AGA CCT TCA AGA GCA TC |
| p65-r | ATC ATA GTT GAT GGT GCT CAG G |
| p65-CDS-f | ATG GAC GAA CTG TTC CCC CTC |
| p65-CDS-r | TTA GGA GCT GAT CTG ACT CAG C |
