# Supplementary figures and images for: MiR-182 promotes cancer invasion by linking RET oncogene activated NF-κB to loss of the HES1/Notch1 regulatory circuit
Source: Mol Cancer. 2017 Jan 26;16:24. doi: 10.1186/s12943-016-0563-x (PMC5267421; doi:10.1186/s12943-016-0563-x)

Figure S1

a

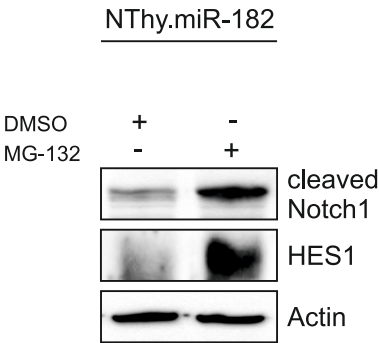

b

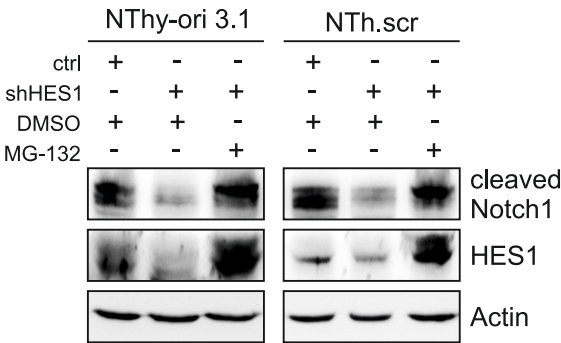

Supplement: Additional file 2: — Figure S1. Inhibition of ubiquitin-dependent degradation of intracellular active Notch1. a-b Effect of inhibitor MG-132 on cleaved Notch1 and HES1 expression inNThy.miR-182 (a), NThy-ori 3.1 and NThy.scr transfected with shHES1 or control (b). Cells were treated with 40 μM MG-132 or equal amount of DMSO for 5 h and at 24 h post transfection. Expression of cleaved Notch1 and HES1 were analyzed by WB. Actin was used as loading control. (PDF 3001 kb) [file 12943_2016_563_MOESM2_ESM.pdf]
